# Macrophages and neutrophils express IFNλs in granulomas from *Mycobacterium tuberculosis*-infected nonhuman primates

**DOI:** 10.3389/fimmu.2022.985405

**Published:** 2022-09-13

**Authors:** Priyanka Talukdar, Beth F. Junecko, Daniel S. Lane, Pauline Maiello, Joshua T. Mattila

**Affiliations:** ^1^ Department of Infectious Diseases and Microbiology, School of Public Health, University of Pittsburgh, Pittsburgh, PA, United States; ^2^ Center for Vaccine Research, School of Medicine, University of Pittsburgh, Pittsburgh, PA, United States; ^3^ Department of Microbiology and Molecular Genetics, School of Medicine, University of Pittsburgh, Pittsburgh, PA, United States

**Keywords:** tuberculosis, granuloma, interferon lambda IFNλ, macrophage, neutrophil

## Abstract

Granulomas are the hallmark of *Mycobacterium tuberculosis* (Mtb) infection. Cytokine-mediated signaling can modulate immune function; thus, understanding the cytokine milieu in granulomas is critical for understanding immunity in tuberculosis (TB). Interferons (IFNs) are important immune mediators in TB, and while type 1 and 2 IFNs have been extensively studied, less is known about type 3 IFNs (IFNλs) in TB. To determine if IFNλs are expressed in granulomas, which cells express them, and how granuloma microenvironments influence IFNλ expression, we investigated IFNλ1 and IFNλ4 expression in macaque lung granulomas. We identified IFNλ expression in granulomas, and IFNλ levels negatively correlated with bacteria load. Macrophages and neutrophils expressed IFNλ1 and IFNλ4, with neutrophils expressing higher levels of each protein. IFNλ expression varied in different granuloma microenvironments, with lymphocyte cuff macrophages expressing more IFNλ1 than epithelioid macrophages. IFNλ1 and IFNλ4 differed in their subcellular localization, with IFNλ4 predominantly localizing inside macrophage nuclei. IFNλR1 was also expressed in granulomas, with intranuclear localization in some cells. Further investigation demonstrated that IFNλ signaling is driven in part by TLR2 ligation and was accompanied by nuclear translocation of IFNλR1. Our data indicate that IFNλs are part of the granuloma cytokine milieu that may influence myeloid cell function and immunity in TB.

## Introduction

Tuberculosis (TB) is caused by *Mycobacterium tuberculosis* (Mtb) and claims approximately 4,400 lives per day (1), leading to at least 1.5 million deaths per year (2). TB is associated with granuloma formation and immune cells in granulomas engage in coordinated activities that limit Mtb replication and dissemination (3). Immune responses in granulomas are highly regulated and infection outcomes depend on the balance between pro- and anti-inflammatory cytokines (4). This balance is maintained across heterogenous granuloma microenvironments where different regions vary by oxygen tension, cytokine milieu, necrotic cell abundance, and Mtb antigen concentration, all of which affect cellular activation states and functions (4–7). When appropriately balanced, granulomas generate sterilizing immunity (8), but deviation from this equilibrium promotes bacterial dissemination, leading to increasingly severe disease (4). The equilibrium defining these outcomes is not well understood but identification of factors that differentiate protective and detrimental outcomes is critically important for understanding TB pathogenesis.

Cytokine-mediated communication regulates granuloma function (4, 7, 9, 10). Type III IFNs (IFNλs) have important antiviral roles (11–14) but their function in bacterial infections is not well characterized. The human genome encodes four IFNλ proteins that are sometimes identified by their interleukin identifier including IFNλ1 (IL-29), IFNλ2 (IL-28A), IFNλ3 (IL-28B), and IFNλ4 (11, 12, 15). IFNλ1-3 have 80-96% amino acid sequence identity (11), whereas IFNλ4 is 28% identical to the other IFNλs. In humans, IFNλ4 is encoded by transcripts with a ΔG allele of a genetic variant rs368234815, while the TT allele, introduces a frameshift and creates a pseudogene that does not encode IFNλ4 (15, 16). In contrast, due to the invariant presence of the rs368234815-ΔG allele, non-human primate (NHP) genomes encode only the functional IFNλ4 and not the pseudogene (15, 17). Chimpanzees and human African hunter gatherer pygmies encode a more active IFNλ4 (E154) that has higher ISG induction and antiviral potentials, whereas majority of humans encode an attenuated version of IFNλ4 due to mutation of a highly conserved amino acid residue (E154K) (18, 19). IFNλs signal through IFNλR, a heterodimeric receptor consisting of IL28Rα (IFNλR1) and IL-10Rβ (11). Ligation of the IFNλR1/IL-10Rβ receptor complex induces STAT1/2 phosphorylation and expression of IFNλ-regulated genes, many of which overlap with type 1 IFN (IFNα/β)-regulated genes (11, 20).

Surprisingly little is known about how IFNλs affect immune function during TB. Mtb-infection induces *IFNλ2* gene expression in the human lung epithelium-like cell line A549, suggesting that mycobacterial antigens or infection may upregulate IFNλ expression by lung epithelia during TB (21). Consistent with this, elevated IFNλ2 concentrations are present in sputum from individuals with active TB, whereas lower amounts are present in Mtb-negative or latently infected individuals (22). Interestingly, after individuals with active TB were treated with anti-mycobacterial drugs, sputum IFNλ2 concentrations decreased to be equivalent to concentrations seen in healthy individuals, a phenomenon noted as early as 7 days post-treatment (22). These studies suggest that IFNλ is upregulated in Mtb infection, but they do not identify which cells express IFNλ in the lungs of infected people, if IFNλ is expressed in granulomas, or if granuloma cells respond to IFNλ.

Here, we investigate unanswered questions of IFNλ biology in TB using granulomas from Mtb-infected cynomolgus macaques. This NHP is a well-established model of human TB and has been used to generate critical insights into TB pathogenesis and disease (23, 24). Like humans, macaques express all four IFNλs, but unlike humans, macaques do not have the rs368234815-TT allele and thus produce IFNλ4 and not the pseudogene (15), thus giving us the ability to investigate this cytokine without being limited by host genotype. We found that granulomas express more IFNλ than uninvolved lung and identified that IFNλ1 and IFNλ4 were expressed by macrophages and neutrophils, with variation in expression patterns across different granuloma microenvironments. Interestingly, IFNλ4 was expressed by numerous cells and was unique in being localized in the nuclei of macrophages. IFNλ stimulation induced IFNλR1 localization to the nuclei of human cell lines, monocyte-derived macrophages from macaques, and epithelial cells and other cells in granulomas, suggesting a relationship between receptor nuclear translocation and signaling *in vitro* and *in vivo*. Our results provide new insight into IFNλ biology in TB and suggest that IFNλs may have unappreciated roles in anti-mycobacterial immunity.

## Materials and methods

### Animal ethics statement and sourcing of macaque tissue samples

Animal procedures and husbandry practices were performed according to protocols approved by University of Pittsburgh’s Institutional Animal Use and Care Committee (IACUC) which adheres to guidelines established in the Animal Welfare Act, Guide for the Care and Use of Laboratory Animals, and Weatherall report (eighth edition). The University of Pittsburgh is fully accredited by the Association for Assessment and Accreditation of Laboratory Animal Care. The tissue sections and samples included in this study originated from animals that were necropsied as part of other studies and made available as convenience samples. Briefly, cynomolgus macaques (*Macaca fascicularis*) were infected with 4-415 CFU of Erdman-strain Mtb *via* intra-tracheal instillation or aerosol inhalation (23, 25). At the end of the study, animals were humanely euthanized and necropsied as described previously (25, 26) and tissues were excised and fixed in 10% neutral buffered saline for histology and immunohistochemistry. Fixed samples were paraffin embedded, cut into 5 μm-thick sections and mounted on SuperFrost Plus slides (Thermo Fisher Scientific, Waltham, MA) by the University of Pittsburgh Medical Center’s *in situ* histology lab. Information on each animal by involvement in this work is included in **Supplementary Tables 1**, **2**.

### Immunohistochemistry and fluorescence imaging

A cyclic IHC process, like that described by Lin et al. (27), was used for multiple rounds of staining on the same formalin-fixed paraffin-embedded (FFPE) tissue section. FFPE sections were deparaffinized in xylenes and 100% ethanol and then antigen retrieval was performed in a buffer containing 20 mM Tris/820 μM EDTA/0.0001% Tween 20 [pH 9.0] using a Retriever (Pick Cell, Waltham, MA) as previously indicated (7). Sections were blocked in 1% BSA/PBS for 30 minutes at room temperature before addition of primary antibodies that were diluted in blocking buffer. The slides were washed 3-4 times with 1xPBS and then incubated for 1 hour with species-specific secondaries, or where multiple antibodies from the same species where used, isotype-specific secondary antibodies conjugated with AF488, AF594, or AF647 (Thermo Fisher Scientific, or Jackson ImmunoResearch Laboratories, West Grove, PA). In all cases, antibodies were diluted in blocking buffer. Following incubation in secondary antibodies, slides were washed with 1xPBS and coverslips were applied using ProLong Gold mounting medium containing DAPI (Thermo Fisher Scientific). The mounting medium was cured for 1-2 hours and then the slides were stored at -20°C until they were imaged. After imaging, the slides were incubated in Copland jars containing Milli-Q water until the coverslip fell off and then washed for 20 minutes under gentle shaking at room temperature. Antibodies were stripped off the tissue sections by repeating the process of antigen retrieval (incubation under pressure in antigen retrieval buffer at 121°C for 20 minutes) and stripping was validated by re-mounting a coverslip and reexamining the slide by microscopy. After stripping, the slides were incubated with blocking buffer and a second round of staining with a different combination of primary and secondary antibodies were applied to the tissue section before a coverslip was mounted with DAPI ProLong Gold and the slide was reimaged.

Tissue sections were first stained to detect macrophage and neutrophil IFNλ1 expression and then stripped to visualize IFNλ4 expression in macrophages and neutrophils. To ensure that our results did not include crosstalk between different rounds of staining for cytokine expression, we used different fluorochromes to visualize and quantify IFNλ1 (AF594) and IFNλ4 (AF488) expression. Moreover, the success of stripping the previous round of anti-calprotectin staining (AF488-stained neutrophils) was confirmed visually before beginning analysis of the sections in the second round of staining. The differential localization of these cytokines was also compared and the results of these analyses are included in the Results section. Staining was performed as previously described (6). Antibodies used for staining tissues included CD11c (clone 5D11, 1:30 dilution; Leica Microsystems, Buffalo Grove, IL), calprotectin/S100A9 (clone MAC387, 1:30 dilution; Thermo Fisher Scientific), polyclonal IFNλ1 (1:30 dilution; R&D Systems, Minneapolis, MN), monoclonal IFNλ4 (clone 4G1, 1:50 dilution; EMD Millipore, Burlington, MA) and polyclonal IFNλR1 (1:50 dilution; Sigma Aldrich, St. Louis, MO). Human and non-human primate IFNλ1, IFNλ4 and IFNλR1 transcripts share greater than 90% nucleotide sequence similarity with each other and therefore we expected the anti-human IFNλ1, IFNλ4 and IFNλR1 antibodies to work in non-human primates. For IFNλ4 staining, a directly labeled conjugate of calprotectin-AF594 was used because both anti-calprotectin and anti-IFNλ4 antibodies were mouse IgG1 antibodies. Zenon direct labeling kit (Thermo Fisher Scientific) was used to conjugate calprotectin with AF594. Granulomas were imaged with a Nikon Eclipse E1000 epifluorescence microscope (Nikon Instruments, Melville, NY) at 20x magnification with illumination provided by SOLA light engine (Lumencor, Beaverton, OR) and images captured with a DS-Qi2 camera (Nikon Instruments). NIS-Elements AR 4.50 software (Nikon Instruments) was used for image capture and setting imaging parameters which were fixed across all the granuloma images. Four color channels, with DAPI as the fourth channel, were acquired for all images. Animals used in IHC are mentioned in **Supplementary Table 2**.

### Image analysis

QuPath version 0.2.1 software (28) was used to measure IFNλ expression and fluorescence intensity in granulomas. For quantifying these metrics, whole granuloma images were loaded into QuPath and the cells were classified as neutrophils and macrophages based on calprotectin and CD11c expression, respectively, using a high threshold to eliminate non-specific background signal and ensure only cells that truly expressed these antigens were being analyzed. The threshold intensity for defining IFNλ signal was based on the isotype control and background staining of each tissue section. After classification of positive and negative signal for each channel, the cells were segmented by QuPath based on DAPI signal and the channel intensity measurements for each cell were recorded. Since our measurements are using mean pixel intensity per cell, which normalizes fluorescence per unit area per cell type, we do not expect the different sizes of cell types to impact the interpretation of the intensity data. For analysis of region-based IFNλ intensities, manual segmentation yielded the most accurate results. For these analyses, at least 100-300 neutrophils at the caseum-epithelioid macrophage interface or in the lymphocyte cuff, and macrophages in the epithelioid macrophage region adjacent to caseum or in the lymphocyte cuff were chosen. After all the annotations were selected, the detection measurements were exported which contained mean measurements of individual channels for each cell, as well as for cell nuclei and cytoplasm. QuPath detections were used in CytoMAP version 1.4.7 (29) to generate the spatial map of IFNλ expression in granulomas.

### BCA protein quantification assay and ELISA

Protein levels in supernatants from homogenized granulomas and non-diseased lung lacking bacterial loads and without granulomas (**Supplementary Tables 1**, **2**) was measured using the Pierce BCA Protein Assay Kit (Thermo Fisher Scientific) according to the manufacturer’s protocol. Samples with detectable protein levels were selected for IFNλ level detection by ELISA using a human IL-29/IL-28B (IFN-lambda 1/3) DuoSet ELISA kit (R&D Systems), and the assay was performed according to the manufacturer’s protocol. For reporting data, the IFNλ content was normalized to micrograms of total input protein.

### Flow cytometry

Non-diseased lung was obtained from Mtb-infected macaques (**Supplementary Table 1**) being necropsied as part of ongoing studies. These tissues were mechanically disaggregated with a Medimachine tissue processor (BD Biosciences, San Jose, CA) and single cell suspensions were stained to detect IFNλR1 expression. Samples were stained for viability (Aqua viability dye, Thermo Fisher Scientific) and surface and intracellular markers according to standardized protocols. The antibody panel for IFNλR1 detection in lung tissue included surface marker staining for IL28RA (an alternate name for IFNλR1; Clone MHLICR2a, BioLegend, San Diego, CA), CD45 (Clone D058-1283, BD Biosciences), CD206 (Clone 19.2, BD Biosciences), CD3 (Clone SP34-2, BD Biosciences, CD20 (Clone 2H7, BD Biosciences), CD14 (Clone MφP9, BD Biosciences), CD11b (Clone ICRF44, BD Biosciences), and intracellular staining for calprotectin (Clone MAC387, Thermo Fisher Scientific) labeled by Zenon labeling was used to identify neutrophils. The gating strategy for tissue cells is shown in **Supplemental Figure 1**. As a gating control and to compare IFNλR1 expression in peripheral blood cells and lung tissue, erythrocytes in an aliquot of autologous peripheral blood were lysed using RBC lysing buffer (BD Biosciences) and the nucleated cells were stained at the same time as the tissue cells with the same antibody cocktail. Specificity of the λFNλR1 antibody was confirmed using isotype and fluorescence-minus one (FMO) controls. Data were acquired with a LSRFortessa flow cytometer (BD Biosciences) and analyzed with FlowJo v10 (BD Biosciences).

### Differentiation of monocyte derived macrophages and cell culture

Monocytes were isolated from macaque peripheral blood mononuclear cells (PBMCs) (**Supplementary Table 1**) and cryopreserved using CellBanker II freezing medium (Amsbio, Cambridge, MA). After thawing, cells were labeled with NHP-specific anti-CD14 beads (Miltenyi Biotec, Auburn, CA) according to manufacturer’s instructions. Isolated monocytes were plated in 12-well flat bottom plates that were coated with Anti-Adherence Rinsing Solution (STEMCELL technologies, Cambridge, MA), at a density of 1-1.5x10^6^ cells/well in RPMI 1640 media (Lonza, Walkersville, MD) supplemented with 20% FBS (Gibco, Grand Island, NY), 1% L-glutamine (Sigma-Aldrich St. Louis, MO), 0.1 mM sodium pyruvate (Gibco), 50 μM 2-mercaptoethanol (Gibco), 0.006 μg/ml GM-CSF (Sigma-Aldrich), 0.01 μg/ml M-CSF (Sigma-Aldrich) and 100 U/ml penicillin-streptomycin (Gibco). Media was changed to RPMI 1640 media supplemented with 10% FBS,1% HEPES (HyClone, Logan, UT), 1% L-glutamine (hereafter referred to as R10) and 1mg/ml penicillin (Alfa Aesar, Haverhill, MA). Monocytes were cultured for 7-10 days for differentiation into macrophages with media change every 3-4 days. For studies using human cell lines, monocyte-like THP-1 and lung epithelium-like A549 cell lines were originally purchased from ATCC (Manassas, VA), and were cultured in RPMI/10% FBS supplemented with 100 U/ml penicillin-streptomycin and 50 µM 2-mercaptoethanol (only in THP-1 cell cultures) for 3-4 days before being subcultured for downstream assays.

### IFNλR1 nuclear localization assay

A549 and MDMs were seeded into 12-well chamber slides (ibidi, Fitchburgh, WI) and stimulated with IFNλ1 (100 ng/ml, Peprotech, Cranbury, NJ), IFNλ4 (100 ng/ml, R&D Systems) and gamma-irradiated Mtb (BEI Resources, Manassas, VA) and incubated at 37°C with 5% CO_2_ for 2 hours. After incubation, cells were fixed and permeabilized with the BD Cytofix/Cytoperm kit (BD Biosciences) and washed with 1xPerm-Wash buffer. Assays with THP-1s were done in round-bottom tubes (Corning, Glendale, Arizona). For the TLR1/2 and TLR4 blocking assays, cells were incubated with 2 µM CU CPT 22 (Tocris Bioscience, Minneapolis, MN) and 20 µM C34 (Tocris Bioscience), respectively, for 30 minutes, before addition of gamma-irradiated Mtb. After incubation with gamma-irradiated Mtb, the cells were fixed and cytospin was performed. Cells were then blocked in 1% BSA/PBS containing AF647-labeled phalloidin (1:40 dilution; Thermo Fisher Scientific) for 30 minutes at room temperature, prior to addition of primary and secondary antibodies diluted in 1xPerm-Wash buffer. Anti-IFNλR1 and fluorochrome-conjugated secondary antibody were used at the same dilution as for the IHC experiments described above. After staining, cells were washed in Perm-Wash buffer and coverslips were applied using Prolong Gold mounting medium containing DAPI (Thermo Fisher Scientific). Slides were imaged with an epifluorescence microscope (Nikon Eclipse E1000) at 60x magnification, and a Nikon camera (DS-Qi2) was used to capture the images as previously described.

### Statistics

GraphPad Prism v9.1 (GraphPad Software, San Diego, CA) was used for statistical analyses. None of our analyses used cross-antibody (IFNλ1 vs IFNλ1) tests to avoid confounding factors introduced by antibody affinity and avidity-related issues. The Shapiro-Wilk test was used to test the normality of all datasets before performing statistical analyses and parametric tests were used for normally-distributed data and non-parametric tests were used for data that did not fit a Gaussian (normal) distribution. P< 0.05 was considered to be statistically significant.

## Results

### IFNλ1/3 are expressed in lung granulomas from Mtb-infected macaques

**Figure 1:**
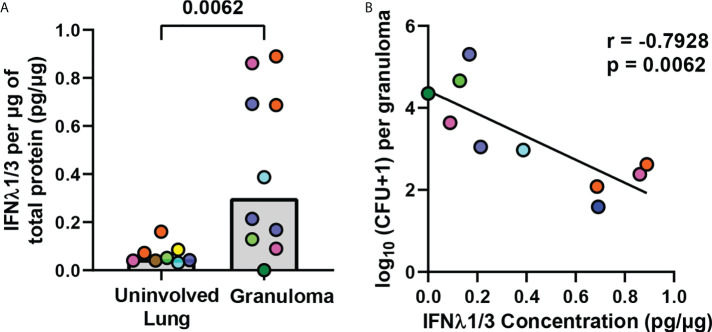
IFNλ1/3 expression in lung granulomas negatively correlates with bacterial burden. **(A)** IFNλ1/3 concentrations in non-diseased uninvolved lung (n=8) and lung granulomas (n=10) were normalized to total protein per sample and compared by ELISA. Bars represent median values. Statistical comparison by Mann-Whitney test for an unpaired comparison. **(B)** Correlation between log_10_ transformed bacterial burden per granuloma and IFNλ1/3 concentration per granuloma. Pearson correlation coefficient and corresponding p-value reported and simple linear regression line shown.

*IFNλ1* and *IFNλ2* genes are upregulated by A549 lung epithelial cells after Mtb stimulation (21) and elevated IFNλ2 protein concentrations are present in sputum from TB patients (22). To determine if IFNλ is expressed in granulomas, we compared IFNλ1/3 protein concentrations in non-diseased lung (no bacteria or granuloma present) and lung granulomas from matched as well as unmatched animals and found significantly more IFNλ1/3 in granulomas than non-diseased lung (**Figure 1A**). Further, a correlation analysis between IFNλ1/3 concentrations and CFU burden in the granulomas revealed a significant negative correlation between IFNλ concentration and CFU/granuloma (**Figure 1B**), suggesting IFNλ1/3 may be associated with improved antibacterial activity. This led us to use IHC to identify cells expressing IFNλ1 in granulomas. We decided to stain for IFNλ1 as it shares greater than 90% similarity at the amino acid level with IFNλ2 and IFNλ3 and is well studied in humans. Importantly, in our preliminary experiments, we also found that the commercially-available reagents for IFNλ1 appeared to work better in NHPs than the reagents we tested for IFNλ2/3 and as a consequence, we continued our follow-up studies by investigating IFNλ1 expression.

**Figure 2:**
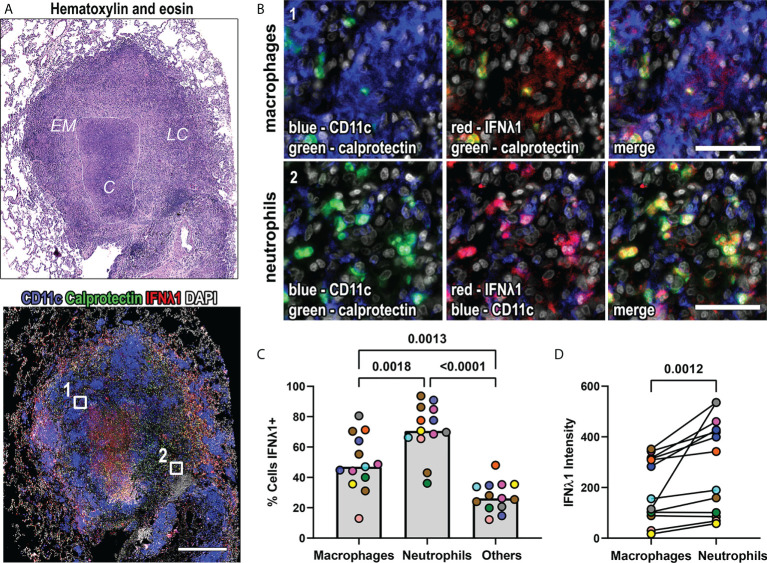
IFNλ1 is expressed in macrophages and neutrophils in granulomas. **(A)** A representative lung granuloma stained with hematoxylin and eosin (H&E; top left) to indicate the lymphocyte cuff (*LC*), epithelioid macrophage (*EM*), and caseous ***(C)*** regions and to detect IFNλ1 (red), CD11c+ macrophages (blue), and calprotectin+ neutrophils (green) (bottom left). Nuclei were stained with DAPI (grey). The white boxes in the immunofluorescence image indicate regions depicted in (B.). Scale bar represents 500 μm. **(B)** Region 1 shows IFNλ1 (red) expression in CD11c+ macrophages (blue). Region 2 shows IFNλ1 (red) expression in calprotectin+ neutrophils (green). Images acquired at 20x magnification, scale bars represent 50 μm. **(C)** Percentage of CD11+ macrophages, calprotectin+ neutrophils, and other cells expressing IFNλ1 in granulomas (n=13). Median values for granuloma are shown where each marker color represents an animal. Statistical comparison by Tukey’s multiple comparisons test. **(D)** IFNλ1 expression, as measured by median fluorescence intensity, by CD11c+ macrophages and calprotectin+ neutrophils (n=13 granulomas). Each point depicts the median intensity values for macrophages or neutrophils per granuloma, with each marker’s color representing a different animal. Statistical comparison by Wilcoxon matched-pairs signed rank test.

We selected thirteen granulomas from nine animals, including five animals that had short-term infections (4 weeks; n=7 granulomas), and four that had long-term infections (26-50 weeks, n=6 granulomas) to assess this. A classical granuloma structure is composed of a central necrotic (caseous) core of necrotic cell debris, surrounded by a layer of epithelioid macrophages, followed by an outer layer referred to as the lymphocyte cuff that contains T and B cells, but also contains macrophages (**Figure 2A**). We used CD11c as a macrophage marker because it is expressed by alveolar and epithelioid macrophages (6, 30), and calprotectin as a neutrophil marker (6). We found that IFNλ1 was expressed by macrophages and neutrophils (**Figure 2B**). We used image analysis to identify the frequency of IFNλ1-expressing macrophages, neutrophils, and the other cells not labeled by our markers. We found that neutrophils were the cell subset most likely to express IFNλ1, followed by macrophages (**Figure 2C**). Further, we measured IFNλ1 intensity/cell as a proxy for IFNλ1 expression by cell type and found that neutrophils expressed significantly more IFNλ1 than macrophages (**Figure 2D**). Overall, these data show that granulomas express higher levels of IFNλ and that macrophages and neutrophils contribute to IFNλ1 expression in granulomas.

### IFNλ1 expression differs by cell type and granuloma microenvironment

**Figure 3:**
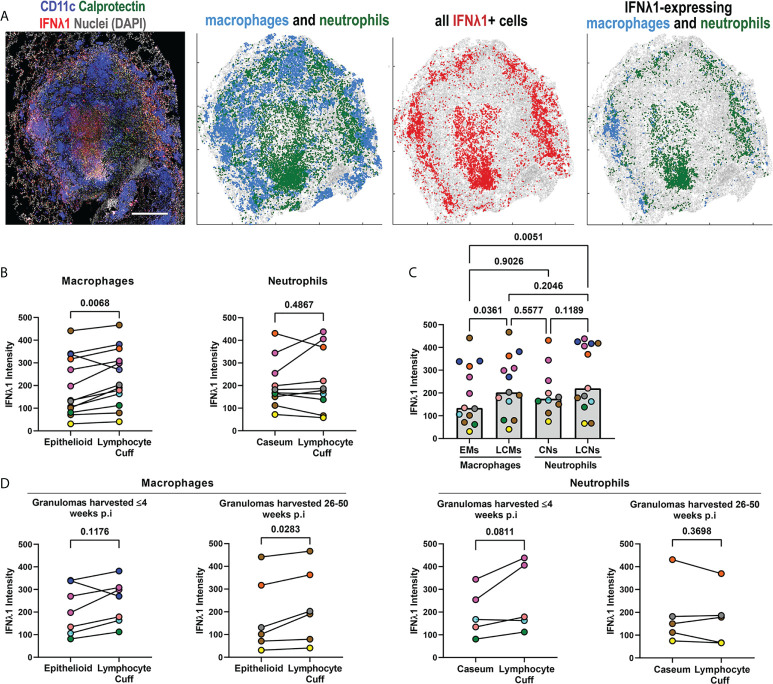
IFNλ1 expression varies by granuloma microenvironment. **(A)** A representative granuloma stained to identify IFNλ1 (red) expressed by CD11c+ macrophages (blue) and calprotectin+ neutrophils (green). Scale bar represents 500 μm. Spatial distribution of macrophages (blue) and neutrophils (green) in the granuloma, distribution of IFNλ1 (red), and distribution of IFNλ1+ macrophages (blue) and neutrophils (green). **(B)** Comparison of IFNλ1 expression, as measured by median fluorescence intensity for each cell subset per granuloma, for epithelioid and lymphocyte cuff macrophages (n=13) (left), and caseum and lymphocyte cuff neutrophils (n=10) (right). Statistical comparisons by paired t test. **(C)** Comparison of median IFNλ1 intensity in epithelioid macrophages, lymphocyte cuff macrophages, caseum neutrophils, and lymphocyte cuff neutrophils (n=13 granulomas). A mixed effect test used to account for repeated measures and pairwise groups compared using Tukey’s multiple comparisons test (Tukey adjusted p-values reported). **(D)** Comparison of IFNλ1 expression, as measured by median fluorescence intensity, between epithelioid and lymphocyte cuff macrophages (left) in granulomas harvested within 4 weeks post-infection (n=7) or 26-50 weeks post-infection (n=6). A similar comparison of IFNλ1 expression by caseum and lymphocyte cuff neutrophils (right) from granulomas harvested by 4 weeks post-infection (n=5) or between 26-50 weeks post-infection (n=5). Statistical comparisons by paired t test.

Granulomas contain unique microenvironments (6) and we performed spatial analyses to identify IFNλ1’s distribution by granuloma region. We found that IFNλ1 was expressed by lymphocyte cuff cells and adjacent to necrotic regions. When the cell types in each region were considered, we found that macrophages and neutrophils in the lymphocyte cuff and neutrophils in necrotic regions expressed IFNλ1 (**Figure 3A**). To investigate differences in IFNλ1 expression by cell type between these regions, we quantified the intensity of IFNλ1 fluorescence by macrophages in the lymphocyte cuff and epithelioid macrophage region and neutrophils in lymphocyte cuff and necrotic regions as a proxy for IFNλ1 protein content (**Figure 3B**). Pairwise comparisons revealed that lymphocyte cuff macrophages expressed significantly more IFNλ1 than epithelioid macrophages whereas neutrophils in lymphocyte cuff and necrotic regions expressed equivalent amounts (**Figure 3B**). We then compared the IFNλ1 intensity across macrophages and neutrophils in these regions and found that epithelioid macrophages expressed less IFNλ1 than lymphocyte cuff neutrophils and macrophages (**Figure 3C**). Since the animals involved in this study were infected for different durations, i.e., some necropsied during early infection (<=4 weeks p.i) and others during late infection (26-50 weeks p.i), we wanted to see if IFNλ1 expression differed in granuloma macrophages and neutrophils from animals with early or late infection. We found greater IFNλ1 expression in lymphocyte cuff macrophages relative to epithelioid macrophages in granulomas harvested later during infection but not early infection (**Figure 3D**). In contrast, there were no significant differences between lymphocyte cuff and caseum neutrophils in granulomas from either infection stage (**Figure 3D**). These data suggest that IFNλ1 expression varies in macrophages from different granuloma microenvironments, which may differentially impact the functions of neighboring cells in the granuloma.

### IFNλ4 is expressed in macaque granulomas

**Figure 4:**
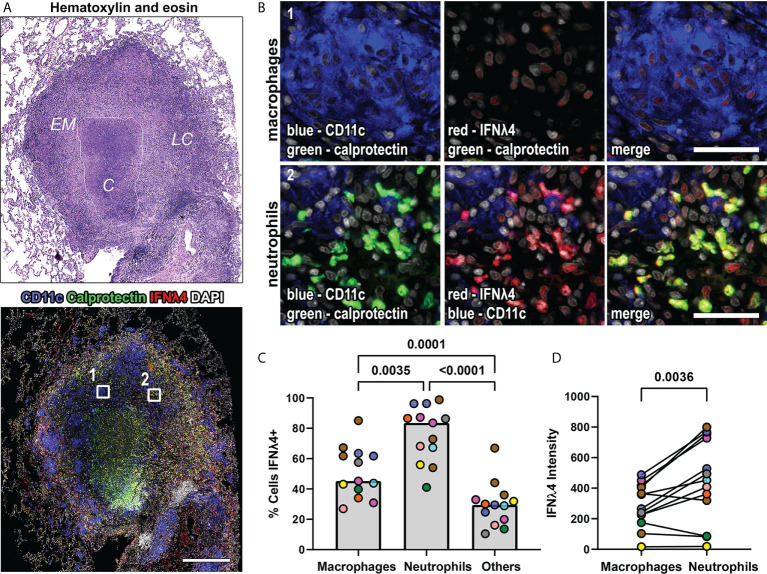
IFNλ4 is expressed in macrophages and neutrophils in granulomas. **(A)** A representative lung granuloma stained with H&E (top left) to indicate the lymphocyte cuff (*LC*), epithelioid macrophage (*EM*), and caseous **(*C*)** regions and to detect IFNλ4 (red), CD11c+ macrophages (blue), and calprotectin+ neutrophils (green) (bottom left). Nuclei were stained with DAPI (grey). The white boxes in the immunofluorescence image indicate regions depicted in **(B)** Scale bar represents 500 μm. **(B)** Region 1 shows IFNλ4 (red) expression in CD11c+ macrophages (blue). Region 2 shows IFNλ4 (red) expression in calprotectin+ neutrophils (green). Images acquired at 20x magnification, scale bars represent 50 μm. **(C)** Percentage of CD11+ macrophages, calprotectin+ neutrophils, and other cells expressing IFNλ4 in granulomas (n=13). Median values for granuloma are shown where each marker color represents an animal. Statistical comparison by Tukey’s multiple comparisons test. **(D)** IFNλ4 expression by CD11c+ macrophages and calprotectin+ neutrophils as measured by median fluorescence intensity by cell subset per granuloma (n=13 granulomas). Each point depicts the median values for macrophages or neutrophils per granuloma, with each marker’s color representing a different animal. Statistical comparison by Wilcoxon matched-pairs signed rank test.

We also investigated IFNλ4 expression, the IFNλ protein with the greatest amino acid sequence divergence from the other IFNλs and found IFNλ4 expression by macrophages, neutrophils, and other granulomas cells (**Figures 4A, B**). In our pilot experiments, we were surprised by the abundance of IFNλ4 in different cell types, and to verify that our IHC-based staining was representative of the overall capacity to express IFNλ4, we used RNAscope with probes against *IFNλ4* mRNA to detect this cytokine’s transcripts *in situ* (**Supplemental Figure 2**). We performed this assay in conjunction with IHC to detect CD163 as an alveolar macrophage marker and found that *IFNλ4* mRNA was detectable in a broad range of cell types in non-diseased lung but was enriched in alveolar macrophages (**Supplemental Figure 2**). This data provided support that our antibody-based detection of IFNλ4 was representative for this protein’s expression, and to accommodate our sample set, we proceeded with IFNλ4 IHC-based staining and analysis of NHP granulomas. To better understand the distribution of IFNλ4+ cells within granuloma macrophages and neutrophils, we quantified the frequency of IFNλ4- expressing cells in FFPE granulomas. We found that calprotectin+ neutrophils were more likely to express IFNλ4 than CD11c+ macrophages, and that macrophages were more likely to express IFNλ4 than non-neutrophil and non-macrophage subsets (**Figure 4C**). Further, pairwise comparison of the intensity of IFNλ4 staining as a proxy for IFNλ4 expression revealed that neutrophils expressed more IFNλ4 than macrophages (**Figure 4D**).

**Figure 5:**
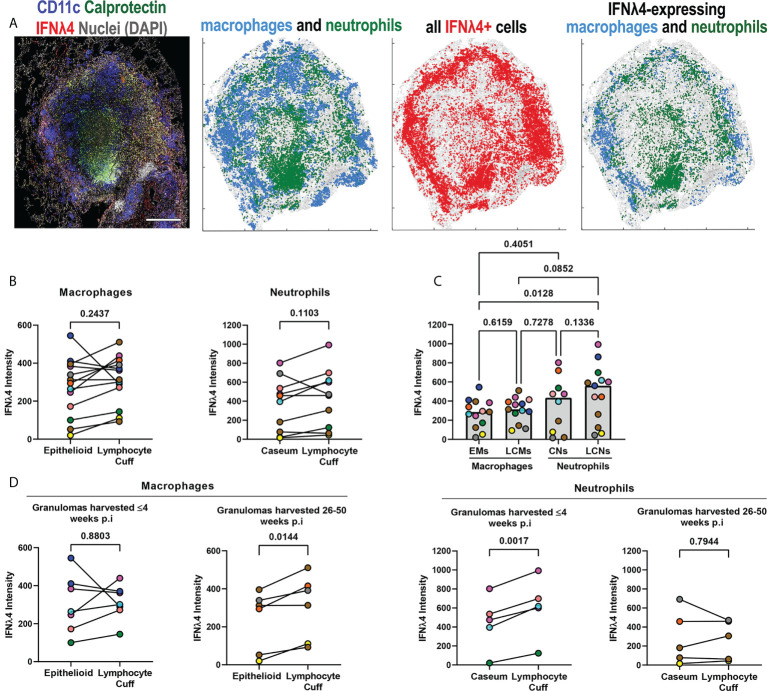
IFNλ4 expression varies by granuloma microenvironment. **(A)** A representative granuloma stained to identify IFNλ4 (red) expressed by CD11c+ macrophages (blue) and calprotectin+ neutrophils (green) (right). Scale bar represents 500 μm. Spatial distribution of macrophages (blue) and neutrophils (green) in the granuloma, distribution of IFNλ4 (red), and distribution of IFNλ4+ macrophages (blue) and neutrophils (green). **(B)** Comparison of IFNλ4 expression, as measured by median fluorescence intensity per cell subset per granuloma, for epithelioid and lymphocyte cuff macrophages (n=13) (left), and caseum and lymphocyte cuff neutrophils (n=10) (right). Statistical comparisons by paired t test. **(C)** Comparison of median IFNλ4 intensity in epithelioid macrophages, lymphocyte cuff macrophages, caseum neutrophils, and lymphocyte cuff neutrophils (n=13 granulomas). A mixed effect test used to account for repeated measures and pairwise groups compared using Tukey’s multiple comparisons test (Tukey adjusted p-values reported). **(D)** Comparison of IFNλ4 expression, as measured by fluorescence intensity, between epithelioid and lymphocyte cuff macrophages (left) in granulomas harvested within 4 weeks post-infection (n=7) or 26-50 weeks post-infection (n=6). A similar comparison of IFNλ4 expression by caseum and lymphocyte cuff neutrophils (right) from granulomas harvested by 4 weeks post-infection (n=5) and between 26-50 weeks post-infection (n=5). Statistical comparisons by paired t test.

Next, we investigated the spatial distribution of IFNλ4 to determine where it was most likely to be expressed by macrophages and neutrophils in granulomas. We observed that IFNλ4 was widely expressed in granulomas, with prominent lymphocyte cuff expression and differences in IFNλ4+ macrophage and neutrophil localization (**Figure 5A**). To identify if macrophage and neutrophil IFNλ4 expression varied by microenvironment, we performed pairwise comparisons on IFNλ4 signal intensity (expression) between macrophages in lymphocyte cuff and epithelioid macrophage regions, and calprotectin+ neutrophils in the lymphocyte cuff and adjacent to caseum. We did not find differences in IFNλ4 expression between spatially-distinct macrophage and neutrophil populations (**Figure 5B**), but a comparison among these cell populations showed lymphocyte cuff neutrophils expressed more IFNλ4 than epithelioid macrophages (**Figure 5C**).

After investigating relative IFNλ4 expression by macrophages and neutrophils in different granuloma regions, we stratified our granulomas by the time point post infection to determine if duration of infection affects IFNλ4 expression. We did not find significant differences between macrophage populations in animals with early-stage disease, whereas lymphocyte cuff macrophages expressed more IFNλ4 than epithelioid macrophages from animals with long-term infections (**Figure 5D**). Interestingly, in examining neutrophil IFNλ4 expression, we found that lymphocyte cuff neutrophils from animals with early-stage TB expressed more IFNλ4 than neutrophils in caseum, whereas differences were not observed in granulomas from animals with later-stage disease (**Figure 5D**). These data indicate that IFNλ4 is expressed in granulomas, primarily by macrophages and neutrophils, and its expression in different cell types can be influenced by the duration of infection.

### IFNλ1 and IFNλ4 differ in their subcellular localization

**Figure 6:**
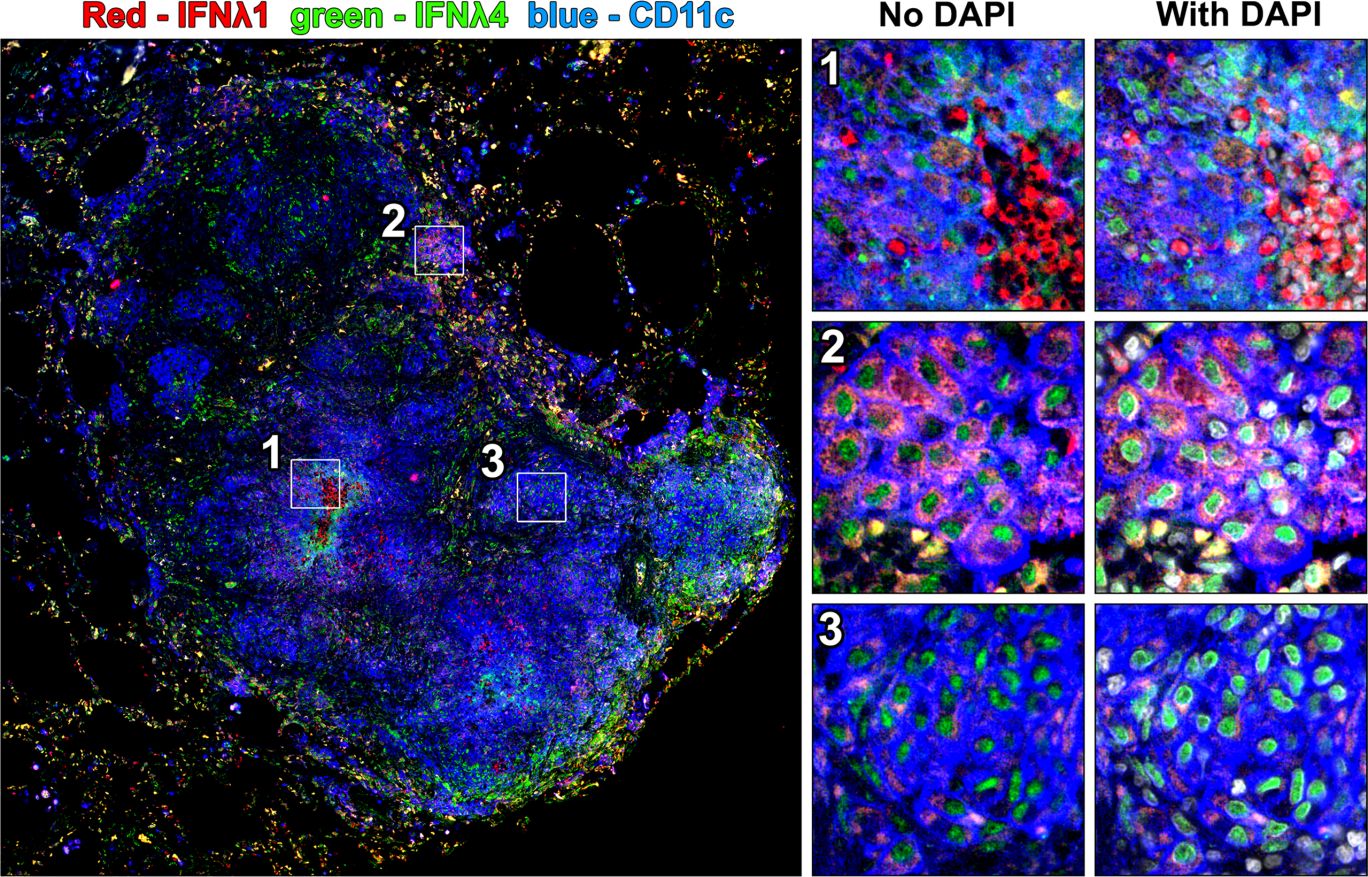
Co-staining for IFNλ1 and IFNλ4 reveals different patterns of expression for these cytokines in granulomas. A granuloma was stained for both IFNλ1 and IFNλ4 and three patterns of IFNλ expression were highlighted including strong cytoplasmic IFNλ1 expression in a cluster of infiltrating neutrophils (region 1), cytoplasmic IFNλ1 and nuclear IFNλ4 expression in lymphocyte cuff macrophages (region 2), and limited cytoplasmic IFNλ1 expression and robust nuclear IFNλ4 expression in epithelioid macrophage-like cells (region 3).

**Figure 7:**
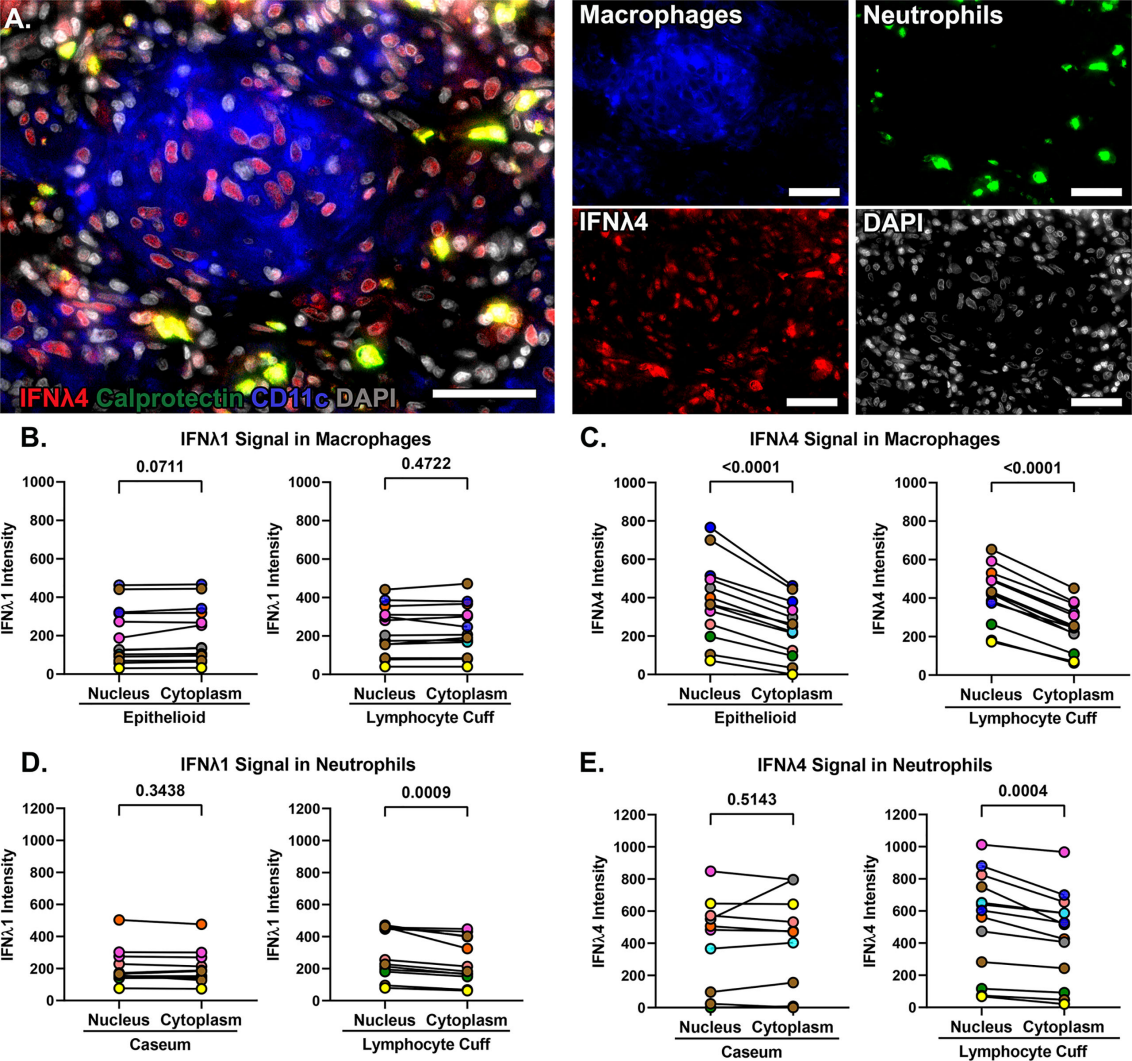
IFNλ1 and IFNλ4 differ in subcellular localization in macrophages. **(A)** IFNλ4 (red) localization in calprotectin+ neutrophils (green) and in the nuclei (grey) of CD11c+ macrophages (blue). 40x magnification, scale bars represent 50 um. **(B)** Comparison of IFNλ1 intensity in the nuclei and cytoplasm of epithelioid (left) and lymphocyte cuff macrophages (right). **(C)** Comparison of IFNλ4 intensity in the nuclei and cytoplasm of epithelioid (left) and lymphocyte cuff macrophages (right). **(D)** Comparison of IFNλ1 intensity in the nuclei and cytoplasm of caseum (left) and lymphocyte cuff neutrophils (right). **(E)** Comparison of IFNλ4 intensity in the nuclei and cytoplasm of caseum (left) and lymphocyte cuff neutrophils (right). In B-E, n=13 granulomas and statistical comparisons done by paired t test.

We noted differences in IFNλ1 and IFNλ4 subcellular localization across cell types. When granulomas were stained with both antibodies simultaneously in conjunction with CD11c as a macrophage marker, we noted different patterns of IFNλ expression in different cell regions including strong localized IFNλ1 expression by infiltrating neutrophils (**Figure 6**, region 1), pockets of alveolar macrophage-like cells in the lymphocyte cuff where cytoplasmic IFNλ1 was co-expressed with nuclear IFNλ4 (**Figure 6**, region 2), and other clusters of macrophages that expressed low levels of cytoplasmic IFNλ1 but stained robustly for IFNλ4 (**Figure 6**, region 3). Interestingly, IFNλ4 was primarily found in DAPI-negative euchromatic regions of macrophage nuclei, while it was more distributed in the cytoplasm of neutrophils (**Figure 7A**). To compare subcellular localization of IFNλ1 and IFNλ4, we segmented the cells and measured each cytokine’s presence in nuclei and cytoplasm. We did not find significant difference between subcellular compartments for IFNλ1 in macrophages from lymphocyte cuff or epithelioid macrophage regions (**Figure 7B**). In contrast, IFNλ4 localized to nuclei rather than cytoplasm of macrophages in both microenvironments (**Figure 7C**). For neutrophils, IFNλ1 and IFNλ4 were present at greater levels in the nucleus of lymphocyte cuff neutrophils, but this difference in subcellular signal intensities was not observed for either cytokine, when the neutrophils were adjacent to caseum (**Figures 7D, E**). We also noted that the difference between nuclear and cytoplasmic signal for IFNλ4 were higher in lymphocyte cuff macrophages (difference in medians = 181.5) and epithelioid macrophages (difference in medians = 116.0), than for lymphocyte cuff neutrophils (difference in medians = 86.6) and neutrophils in the caseum (difference in medians = 21.9). Overall, these data highlight that despite belonging to the same family, IFNλ1 and IFNλ4 have different subcellular localization in macrophages, suggesting they may regulate different cell functions or behaviors.

### Detection of IFNλR1 in NHP lung granulomas

**Figure 8:**
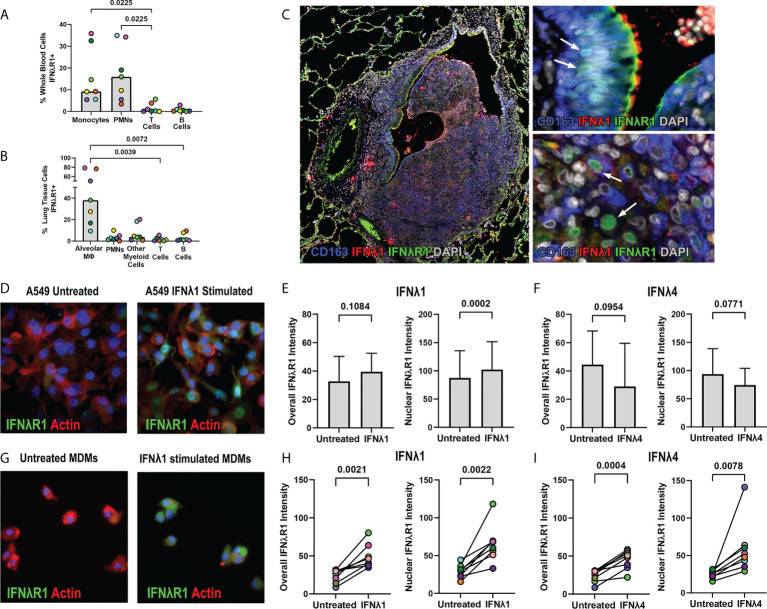
IFNλR1 localizes to the nuclei of macrophages and epithelial cells in granulomas. **(A)** IFNλR1 expression in different immune cell types from whole blood. **(B)** IFNλR1 expression in immune cells from macaque lung tissue (n=7). Friedman test was performed and pairwise groups compared using Dunn’s multiple comparisons test (Dunn’s adjusted p-values are reported). **(C)** Detection of IFNλR1 (green), IFNλ1 (red) and CD163 (blue) in a lung granuloma. Arrows indicate instances of IFNλR1 localized to nuclei. **(D)** A549 lung epithelial cells stained for IFNλR1 (green), actin (red) and DAPI (blue) after IFNλ1 stimulation. **(E, F)** Comparison of overall (left) and nuclear (right) IFNλR1 intensity in A549 epithelial cells, following IFNλ1 **(E)** and IFNλ4 **(F)** stimulations. Graphs show the mean value and standard deviation of 8 independent assays. Statistical comparisons by paired t test. **(G)** MDMs stained for IFNλR1 (green), actin (red) and DAPI (blue) after IFNλ1 stimulation. **(H, I)** Comparison of overall (left) and nuclear (right) IFNλR1 intensity in monocyte-derived macrophages, following IFNλ1 **(H)** and IFNλ4 **(I)** stimulations (n=8). Each point depicts the median IFNλR1 value in macrophages, with each marker’s color representing a different animal. Statistical comparisons by paired t test **(H)** and paired t test for overall IFNλR1 intensity or Wilcoxon matched-pairs signed rank test for nuclear IFNλ4 intensity **(I)**.

Since we identified IFNλ expression in granulomas, we next wanted to identify IFNλR1 expression to determine if granuloma cells can respond to IFNλ. In preliminary work using flow cytometry to measure IFNλR1 in peripheral blood, we found that myeloid cells including monocytes and neutrophils were more likely to express IFNλR1 than T cells and B cells (**Figure 8A**). To determine if this pattern continued in lung tissue, we stained non-diseased lung tissues from the same animals and found that CD206+ alveolar macrophages were more likely to express IFNλR1 than other immune cells (**Figure 8B**). To refine our understanding of granuloma IFNλR1 expression, we stained FFPE sections for IFNλR1, IFNλ1, and CD163 as a macrophage and ciliated epithelium marker (6, 31). In a section where a granuloma was invading an airway and was adjacent to ciliated epithelia, which would be anticipated to express IFNλR1, we noted strong IFNλR1 expression on the apical surface of ciliated epithelial cells (**Figure 8C**). Interestingly, we also observed IFNλR1 localizing to the nuclei of some epithelial cells and macrophage-like cells (**Figure 8C**) suggesting that IFNλR1 may translocate to the nucleus as has been observed for other IFN receptors (32–34).

To determine if IFNλ signaling is associated with IFNλR1 translocation to the nucleus, we performed *in vitro* experiments measuring IFNλR1 dynamics in human cell lines and macaque monocyte-derived macrophages (MDMs). Stimulation of A549 cells with IFNλ1 induced IFNλR1 translocation from the periphery into the nucleus (**Figure 8D**, **Supplemental Figure 3**). While we observed only a trend of increased overall IFNλR1 signal in A549 after IFNλ1 stimulation, the nuclear IFNλR1 signal intensity was significantly elevated (**Figure 8E**). However, we did not observe significant changes in IFNλR1 dynamics in IFNλ4-stimulated A549 cells (**Figure 8F**). In MDMs, however, both IFNλ1 and IFNλ4 induced significant increases in both overall and nuclear IFNλR1 intensities (**Figures 8G-I**, **Supplemental Figure 3**) indicating this behavior occurs in response to diverse members of this cytokine family in macrophages.

**Figure 9:**
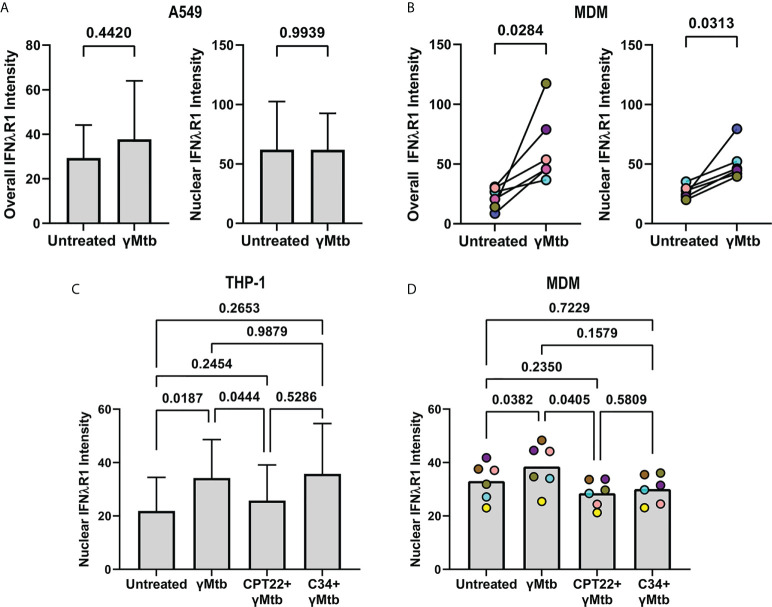
TLR2 mediated signaling by gamma-irradiated Mtb regulates IFNλR1 expression and localization in myeloid cells. **(A)** Comparison of overall (left) and nuclear (right) IFNlR1 intensity in gamma-irradiated Mtb-stimulated A549 epithelial cells. Bars and lines represent the mean value and standard deviation from 5 independent experiments with statistical comparisons by paired t test. **(B)** Overall (left) and nuclear (right) IFNλR1 intensity in gamma-irradiated Mtb-stimulated macaque monocyte derived macrophages (MDMs; n=6). Each point depicts the median IFNλR1 value in macrophages, with each marker’s color representing a different animal. Statistical comparisons by Wilcoxon matched-pairs signed rank test and paired t test, respectively. **(C)** Comparison of nuclear IFNλR1 intensity in gamma-irradiated Mtb stimulated THP-1 cells with or without CU CPT22 (TLR2 antagonist) and C34 (TLR4 antagonist). Bars and lines represent mean values and standard deviation of 7 independent experiments. RM one-way ANOVA used to account for repeated measures and pairwise groups compared using Tukey’s multiple comparisons test (Tukey’s adjusted p-values reported). **(D)** Comparison of nuclear IFNλR1 intensity in gamma-irradiated Mtb-stimulated macaque MDMs with or without CU CPT22 (TLR2 antagonist) and C34 (TLR4 antagonist) (n=6). Each point depicts the median IFNλR1 value for an animal’s MDMs, with each marker’s color representing a different animal. RM one-way ANOVA used to account for repeated measures and pairwise groups compared using Tukey’s multiple comparisons test (Tukey’s adjusted p-values reported).

We next wanted to investigate if Mtb antigens induce IFNλR1 translocation as an indicator of IFNλ signaling. Stimulating A549 cells with gamma-irradiated Mtb did not significantly upregulate overall or nuclear IFNλR1 expression (**Figure 9A**), whereas gamma-irradiated Mtb-stimulated MDMs had increased overall and nuclear IFNλR1 expression (**Figure 9B**). We previously demonstrated that neutrophil cytokine expression could be antagonized by inhibiting toll like receptor (TLRs) signaling (7), so next, we sought to determine how antagonizing TLRs affect nuclear translocation of IFNλR1. Myeloid cells responded more strongly than A549 cells, so we used the human monocyte-like THP-1 cell line in our initial experiments and compared nuclear IFNλR1 localization after inhibition of TLR signaling by the TLR1/2 and TLR4 antagonists CU CPT22 and C34, respectively. We found that CU CPT22, but not C34, inhibited Mtb-mediated nuclear IFNλR1 translocation in THP-1 cells (**Figure 9C**). We observed a similar and significant decrease in nuclear IFNλR1 intensity when MDMs were treated with CU CPT22 (**Figure 9D**), suggesting that IFNλ expression and signaling in myeloid cells is at least partially regulated by TLR1/2 signaling. Overall, our data suggest that like type I and II IFNs, IFNλ signaling can include nuclear translocation of IFNλR1 and that Mtb antigens can activate the TLR1/2 pathway in myeloid cells, potentially leading to IFNλ-mediated responses in granuloma cells.

## Discussion

IFNλs are regulators of innate immunity in the lungs (35). Many studies have focused on viral infections where IFNλ is expressed by epithelial and myeloid cells at mucosal surfaces (13, 35). Like the type 1 IFNs, IFNλ expression is triggered by detection of microbe-associated molecular patterns through pattern recognition receptors (36). Bacterial ligands including lipopolysaccharide and agonists of TLR1/2, TLR4, TLR5 and TLR9 can also induce IFNλ expression (37–39). IFNλs have received little attention in host responses to Mtb infection to this point aside from data from Mtb-infected A549 lung epithelial cells (21) and the sputum from TB patients (22). However, the presence and source of IFNλ expression in granulomas has remained undefined. Here, we investigated two IFNλs, IFNλ1 and IFNλ4, to determine if they contribute to a granuloma’s cytokine milieu. Of the four IFNλs, IFNλ1 is the best studied in humans and shares a high degree of amino acid sequence similarity with IFNλ2 and IFNλ3, and so we selected it as a representative member of the IFNλ family. In contrast, IFNλ4 is less conserved at the nucleotide and amino acid level, and its expression has been selected against in both NHPs and humans (15, 40). Our findings demonstrate that IFNλ1 and IFNλ4 are expressed in granulomas but differ in some aspects of their biology, suggesting they have previously unappreciated functions in TB.

Microenvironment-specific cytokine expression may influence a granuloma’s ability to control bacteria. We observed differences in IFNλ expression across granuloma microenvironments and showed that macrophage subsets in the lymphocyte cuff region were more likely to express IFNλ1 than epithelioid macrophages. The factors that drive IFNλ1 expression in different granuloma regions remain to be determined but local cytokine expression and exposure to damage-associated molecular patterns may contribute to IFNλ regulation. Moreover, as previously mentioned, IFNλ expression can be modulated by TLR recognition of microbial products and Mtb antigens may also induce IFNλ expression. IFNλ1 has Th1 skewing properties (41–43) and elevated IFNλ1 expression by lymphocyte cuff macrophages may promote Th1 polarization in T cells, thus promoting macrophage activation and control of bacilli. Relatedly, our ELISA data suggested a negative correlation between IFNλ and granuloma bacteria loads, supporting this possibility. We also observed that lymphocyte cuff macrophages expressed more IFNλ4 than epithelioid macrophages, primarily in granulomas from animals with long-term Mtb infection. Less is known about IFNλ4’s function in immunity, and while specific polymorphic IFNλ4 genotypes are associated with liver fibrosis in chronic hepatitis C infection (44, 45), it remains to be determined if IFNλ4 promotes fibrosis in pulmonary TB. Taken together, we hypothesize that IFNλ expression is likely to be related to a cell’s activation state and the differences we found in region-specific macrophage IFNλ expression highlight variation in macrophage functional capacity across microenvironments characterized by different immunologic and microbiologic stimuli and suggest new routes by which macrophages may engage with neighboring cells.

Neutrophils are often found in granulomas where they are associated with poor outcomes (46–48) but are also linked to protection in some settings (49, 50). We previously showed that neutrophils express cytokines in granulomas (7), and our work here extends that to expression of IFNλ1 and IFNλ4. Interestingly, neutrophils appeared to be a major population expressing IFNλ in granulomas, producing comparatively higher levels of IFNλs than macrophages. Unlike macrophages, neutrophils in different granuloma microenvironments expressed almost equivalent levels of IFNλs. IFNλs have been identified as critical regulators of neutrophil functions, since they can activate as well as inhibit neutrophil effector functions (51, 52). However, IFNλ expression by neutrophils has not been thoroughly investigated and the protective or pathologic implications for neutrophil-produced IFNλ in TB remain unclear.

Our work highlights novel aspects of IFNλ biology in tissue including protein localization and receptor dynamics. We noted not just cytoplasmic presence of IFNλ, but also intranuclear localization. Intranuclear localization of IFNλ4 was particularly prominent, especially in macrophages, and is attributable to IFNλ4’s nuclear localization signal (NLS) (53). The relevance of this feature is not well understood but intranuclear localization is reported for other IFNs including IFNγ where nuclear translocation of complexed IFNγ-IFNγR enhances IFNγ’s biologic activities (33, 54). Interestingly, IFNλ4 was abundant in neutrophil cytoplasm, which differed from other cells in granulomas. The reasons underlying this are unclear, but this distinction may have implications for a neutrophil’s ability to secrete and respond to IFNλ4. IFNλ1 was also noted in the nucleus of some cells, albeit at a lower frequency and abundance, further suggesting that this cytokine has different properties than IFNλ4. Not only do cells in granulomas express IFNλ, but some undergo IFNλ-regulated signaling as suggested by nuclear localization of IFNλR1 subunit in some granuloma cells. Nuclear translocation of type I and type II IFN receptor subunits has been reported previously (32–34). The C-terminus of IFNγ contains an NLS that mediates the nuclear translocation of the α subunit of IFNγR, where the ligand-receptor complex acts as a nuclear chaperone for STAT1α transcription factor (33). Similarly, the IFNαR1 subunit contains an NLS and is translocated to the nucleus upon ligand stimulation (34). It needs to be further investigated if IFNλR1 nuclear translocation leads to interaction with any transcription factors or how it affects IFNλ-regulated functions, but our work suggests it may be an important contributor to IFNλ signaling in granulomas.

Our work provides insight into IFNλ as a player in the granuloma cytokine milieu. We found a negative correlation between IFNλ concentration and granuloma bacterial burden, suggesting that IFNλ may be associated with protection in TB. Moreover, we found an unexpected distribution of IFNλ expression in different myeloid cells and future work investigating how IFNλ promotes macrophage anti-Mtb activity, or if IFNλ expression correlates with a different protective factor, will help define the role of this cytokine family in granuloma function.

## Limitations of the study

Our data provides insight into the expression of IFNλs in TB granulomas from NHPs. In the work presented here, we made significant use of IHC on convenience samples and future studies will include evaluation of a larger and more diverse sample set. Moreover, our ability to perform high-dimensional flow cytometry-based experiments was limited by a lack of commercially-available anti-IFNλ antibodies for this application and the lack of mechanically-homogenized granulomas with sufficient macrophage populations for analysis. Although we selected the best-available candidate antibodies for IHC, the anti-human anti-IFNλ1 and IFNλ4 antibodies have not been fully assessed in humans and could have enhanced non-specific binding in the context of macaque tissues. Future work on in-depth characterization of the binding properties of these antibodies to human/NHP proteome and development of better antibodies will improve the interpretation of our data. Moreover, although these antibodies against human proteins cross-reacted with NHP proteins, we recognize that there may be different levels of avidity and affinity for their target proteins. This limited our ability to directly compare IFNλ1 and IFNλ4 expression and we only made direct comparisons with the same antibody and did not make cross-antibody comparisons. Lastly, granuloma macrophages are diverse and here we grouped them into two broad categories based on their location in the granuloma, but there may be variations in IFNλ biology that our experimental design cannot capture because of the limitations we faced in the surface markers we chose. Relatedly, we used CD11c as a broadly-expressed macrophage marker but recognize that this antigen can be expressed by other cell types, including dendritic cells; thus, a subset of our CD11c results may include data from these cell types. Future experiments targeting better-defined populations of macrophages may lead to additional data on the role that IFNλs play in TB granulomas. Data availability statement The raw data supporting the conclusions of this article will be made available by the authors, without undue reservation.

## Data Availability Statement

The raw data supporting the conclusions of this article will be made available by the authors, without undue reservation.

## Ethics statement

The animal study was reviewed and approved by University of Pittsburgh Institutional Animal Care and Use Committee.

## Author contributions

The studies were designed and planned by PT and JM. PT and BJ performed experiments. DL performed the RNAscope experiment verifying IFNλ4 expression by lung cells. PT and JM performed the data analyses and consulted with PM on statistical analyses. All authors contributed to the article and approved the submitted version

## Funding

This project was supported in part by National Institutes of Health grants AI134183, AI118195, Pitt HIV-TB Research and Training Program in India D43TW010039 and startup funding from University of Pittsburgh School of Public Health.

## Acknowledgments

We gratefully acknowledge JoAnne Flynn for providing the macaque samples and Carolyn Bigbee, Cassaundra Ameel, the Flynn Lab veterinary staff for technical assistance. The following reagent was obtained through BEI Resources, NIAID, NIH: *Mycobacterium tuberculosis*, Strain H37Rv, Gamma-Irradiated Whole Cells, NR-49098.

## Conflict of interest

The authors declare that the research was conducted in the absence of any commercial or financial relationships that could be construed as a potential conflict of interest.

## Publisher’s note

All claims expressed in this article are solely those of the authors and do not necessarily represent those of their affiliated organizations, or those of the publisher, the editors and the reviewers. Any product that may be evaluated in this article, or claim that may be made by its manufacturer, is not guaranteed or endorsed by the publisher.
