# Supplementary figures and images for: Macrophages and neutrophils express IFNλs in granulomas from Mycobacterium tuberculosis-infected nonhuman primates
Source: Front Immunol. 2022 Sep 13;13:985405. doi: 10.3389/fimmu.2022.985405 (PMC9516334; doi:10.3389/fimmu.2022.985405)

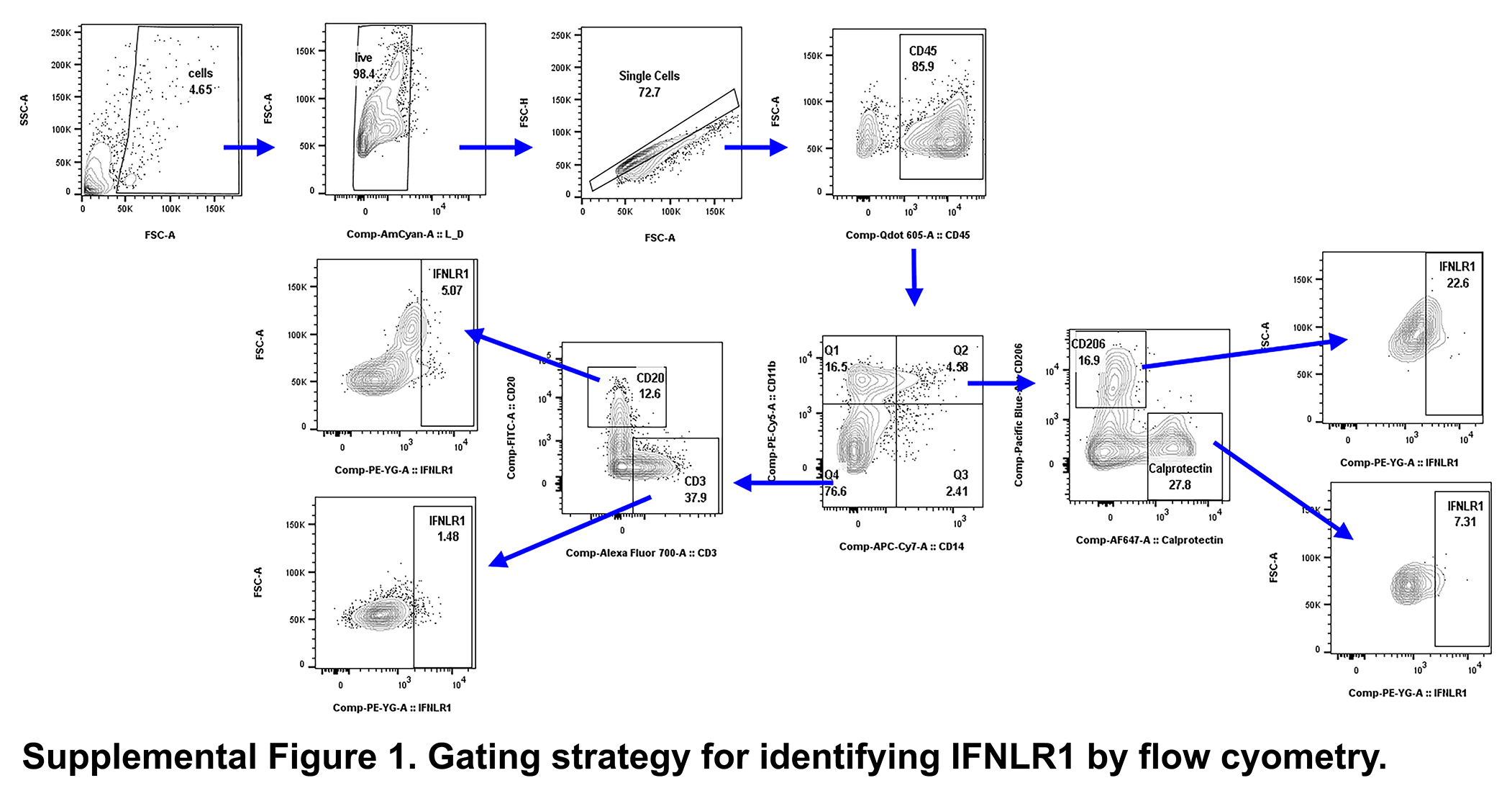

Supplement: Supplementary file 1 [file Image_1.tif]

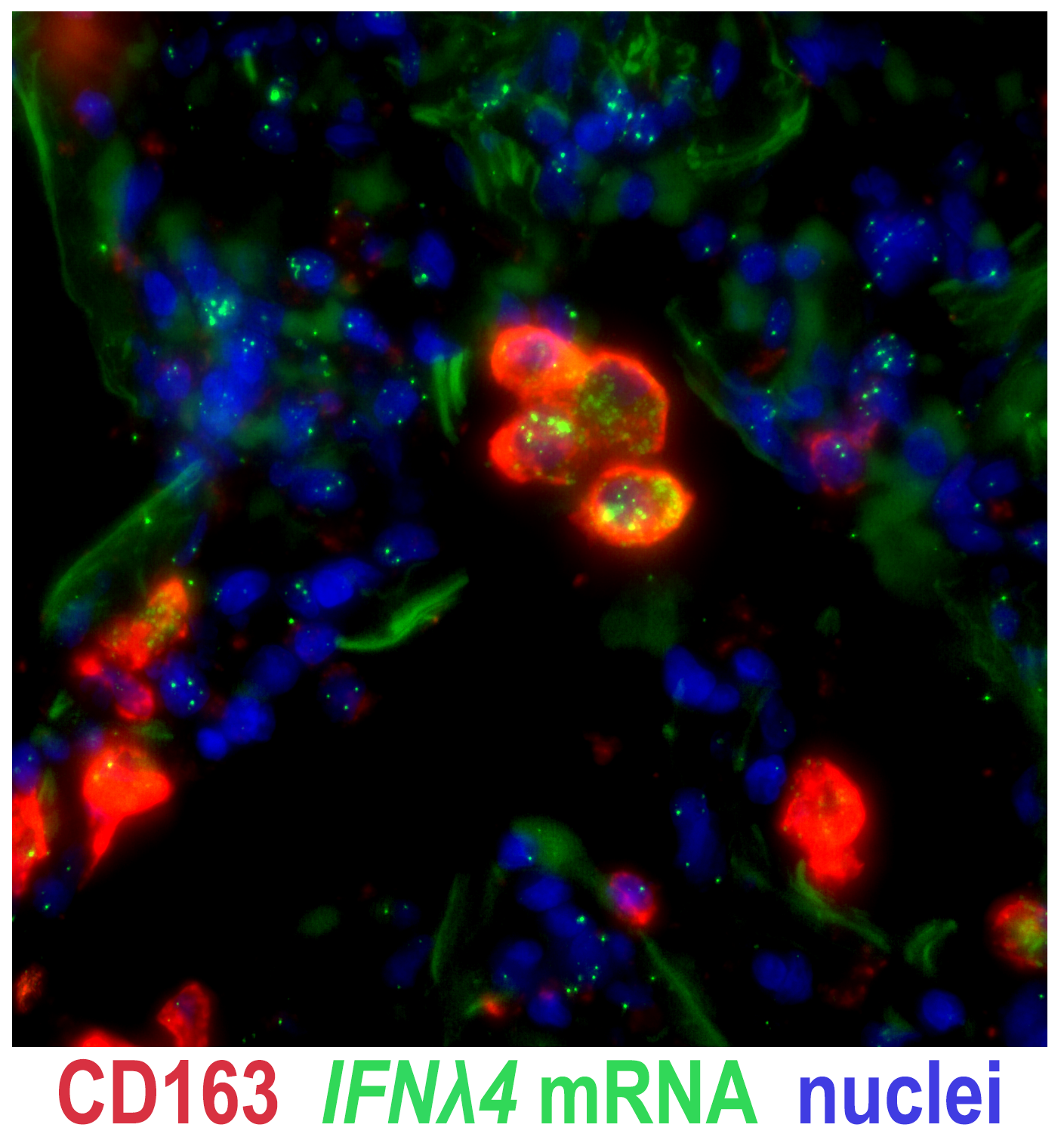

Supplement: Supplementary file 2 [file Image_2.tif]

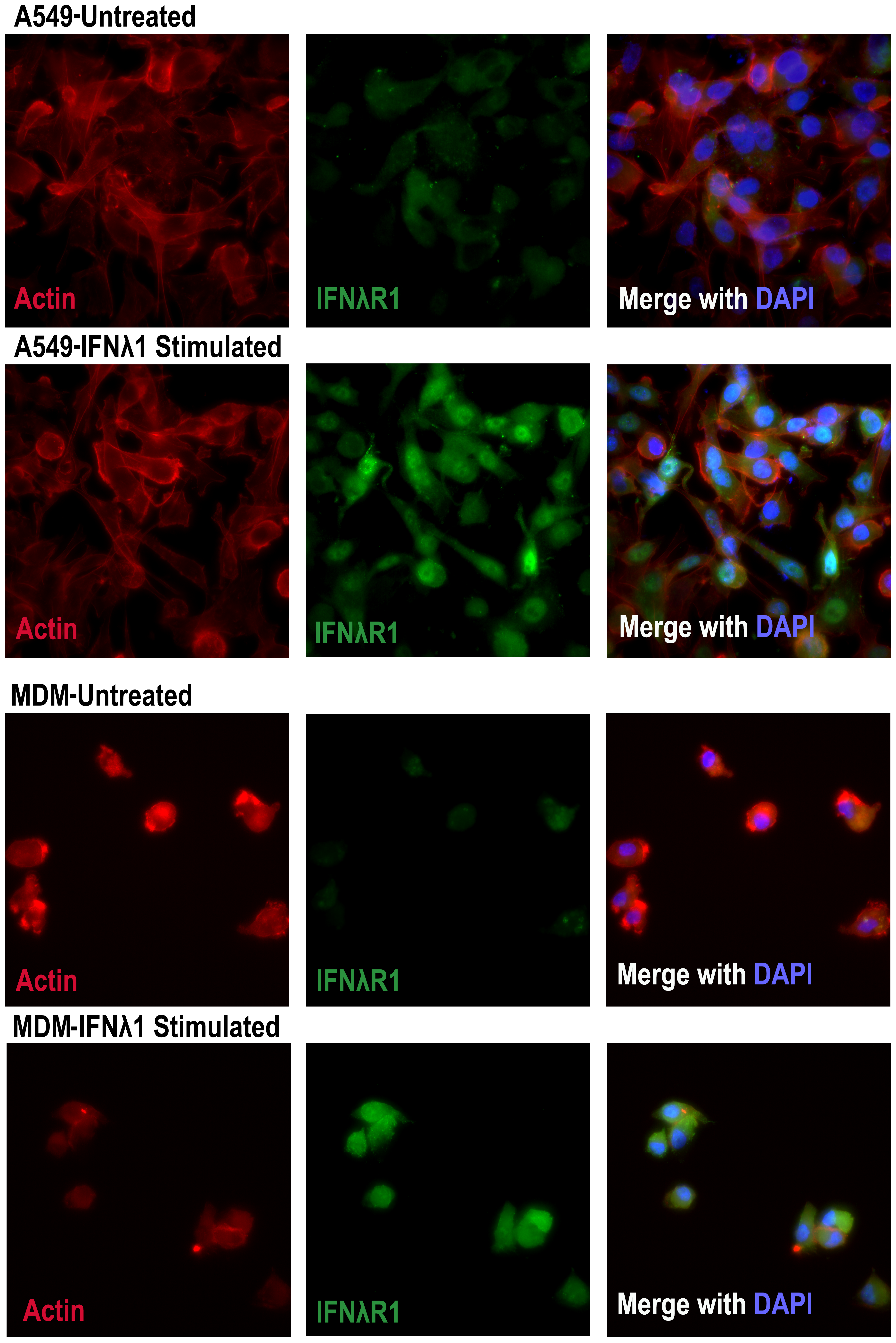

Supplement: Supplementary file 3 [file Image_3.tif]
